# Intestinal adenocarcinoma originating from an undiagnosed Meckel’s diverticulum

**DOI:** 10.1093/jscr/rjac128

**Published:** 2022-05-22

**Authors:** Daniel R Principe, Peter Nesper, Anastasia E Metropulos, Jonathan Rubin, Marin N Marinov

**Affiliations:** Department of Surgery, University of Illinois at Chicago, Chicago, IL, USA; Medical Scientist Training Program, University of Illinois College of Medicine, Chicago, IL, USA; Chicago Medical School at Rosalind Franklin University of Medicine and Science, North Chicago, IL, USA; Northwestern University Feinberg School of Medicine, Chicago, IL, USA; University of Illinois Metropolitan Group Residency Program, Chicago, IL, USA; Chicago Medical School at Rosalind Franklin University of Medicine and Science, North Chicago, IL, USA; Department of Surgery, Advocate Medical Group Lutheran General Hospital, Park Ridge, IL, USA

**Keywords:** Adenocarcinoma, Meckel’s diverticulum, Emergency surgery, GI surgery

## Abstract

Meckel’s diverticulum is a congenital anomaly leading to the formation of a true diverticulum in the distal small intestine. Though most are asymptomatic and discovered incidentally, Meckel’s diverticuli can give rise to a wide range of symptoms. Rarely, this can be a malignancy, most commonly a carcinoid tumor. Other cancers have also been reported, with adenocarcinomas being particularly rare. Here, we report the case of a 62-year-old man presenting to the emergency room with vague gastrointestinal symptoms. Subsequent workup revealed a 3 cm mass in the distal jejunum/proximal ileum, which was located within a previously undiagnosed Meckel’s diverticulum. The mass was sent to pathology, who confirmed an adenocarcinoma arising from a small bowel diverticulum. This case serves as an important reminder of the malignant potential of a Meckel’s diverticulum and adds to the ongoing discussion regarding whether prophylactic diverticulectomy should be recommended to patients with a known Meckel’s diverticulum.

## INTRODUCTION

Meckel’s diverticulum is the most common congenital abnormality of the gastrointestinal (GI) tract and is caused by the incomplete obliteration of the vitelline duct during the fifth week of fetal development [1, 2]. Affecting between 0.3 and 2.9% of the general population, most Meckel’s diverticuli are often clinically silent, though anywhere from 4 to 40% of patients can develop associated symptoms including diverticulitis, hemorrhage and obstruction [3, 4]. Very rarely, patients with a Meckel’s diverticulum can develop a malignancy emanating from the diverticulum, usually in the form of a carcinoid tumor [5, 6]. Meckel’s diverticuli can give rise to other cancers including leiomyosarcomas, peripheral nerve sheath tumors and GI stromal tumors, though these are less common. Adenocarcinomas are exceedingly rare, accounting for as few as 6% of Meckel’s diverticulum-associated malignancies [7]. For patients with a Meckel’s diverticulum-associated adenocarcinoma, prognosis is generally very poor [5]. This is largely attributed to late detection [5], complicated by limited understanding of the disease etiology due to the relatively small number of reported instances.

## CASE PRESENTATION

**Figure 1:**
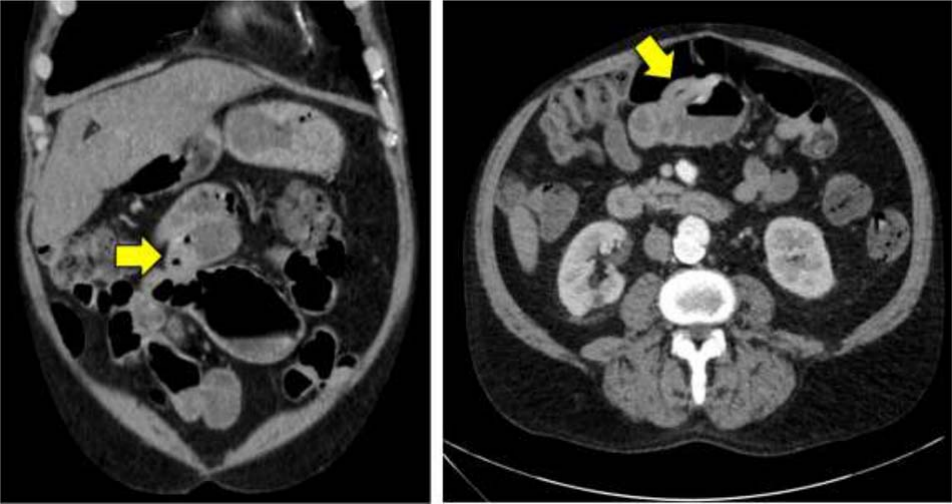
Computed tomography imaging of the abdomen and pelvis showing mild thickening of the wall of the proximal ileum.

A 62-year-old man presented to the emergency room complaining of progressive fatigue, weakness and black stools for 7 days. The patient denied any unintended weight loss, night sweats, headaches, rectal bleeding, bloody stools, hematemesis or other related symptoms. His medical history was significant for a previous hemorrhagic stroke without any neurological deficits as well as an open abdominal aortic repair, and he had no family history of malignancy. Physical exam was largely unremarkable. The patient was afebrile, hemodynamically stable, and his abdomen was soft and non-tender with no palpable masses. Similarly, rectal exam did not reveal any obvious pathology, though hemoccult test was positive. Laboratory studies revealed a hemoglobin of 7.1 g/dl (normal range 13.5–17.5 g/dl) with mean corpuscular volume of 75.9 fl (normal range 80–100 fl), consistent with a microcytic anemia. A subsequent computed tomography angiogram of the abdomen and pelvis was performed, which was only significant for mild thickening of the wall of the proximal ileum (Fig. 1).

**Figure 2:**
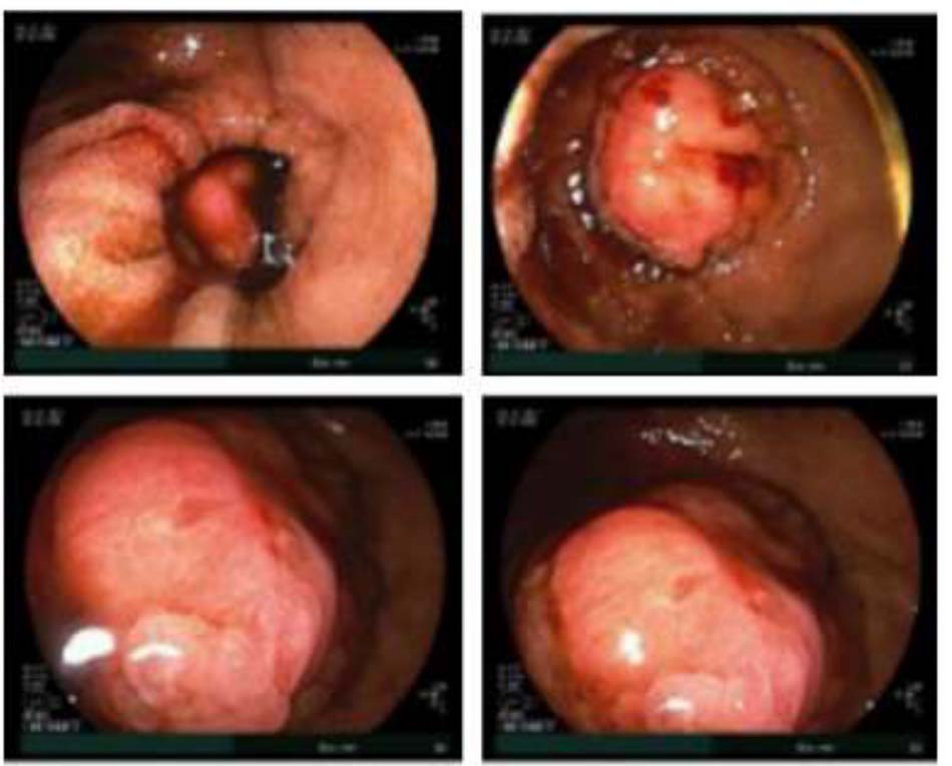
Double balloon enteroscopy revealing a 3 cm mass located in the distal jejunum/proximal ileum.

**Figure 3:**
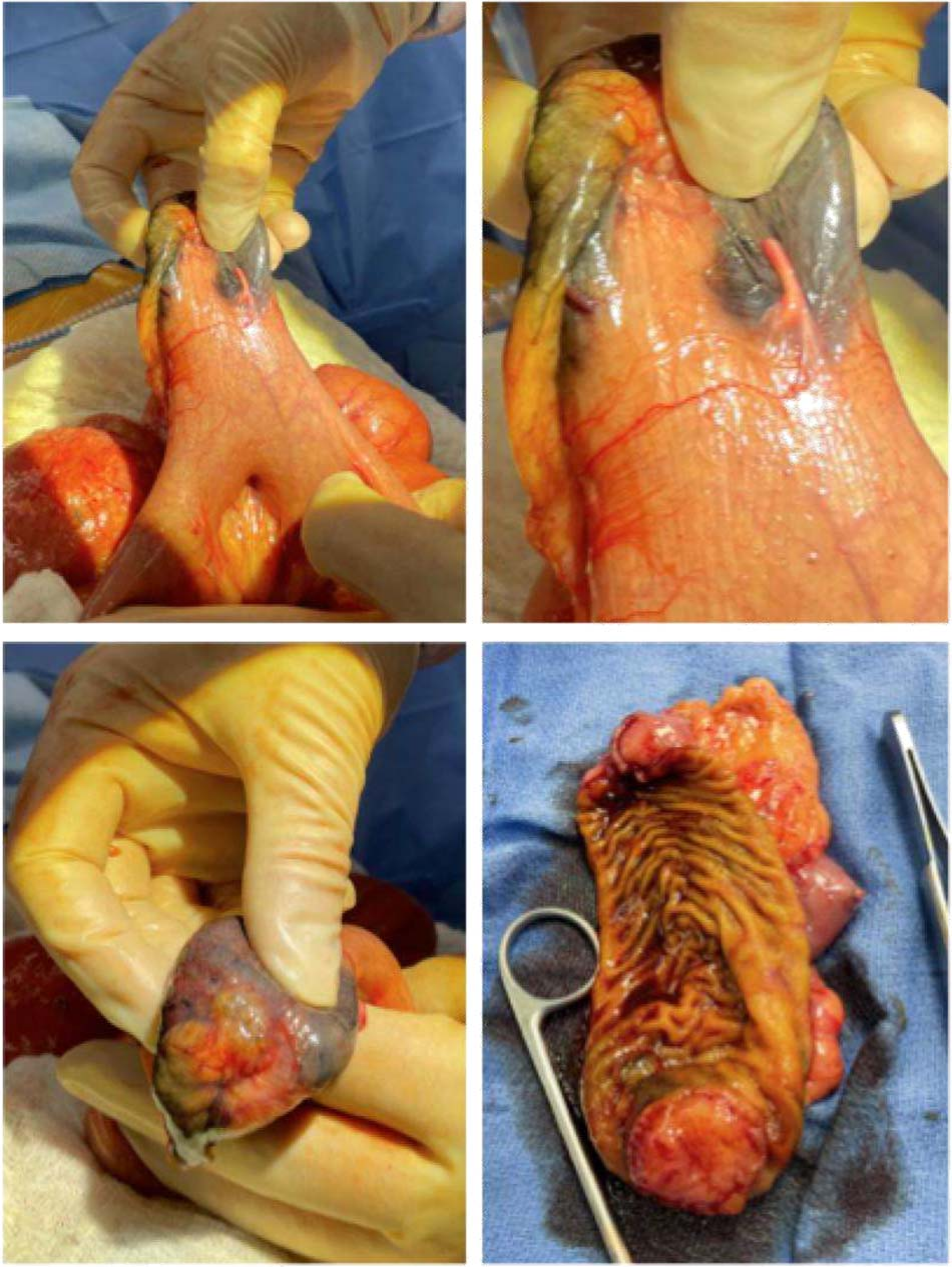
Gross pathology images of the bowel mass located within a Meckel’s diverticulum.

**Figure 4:**
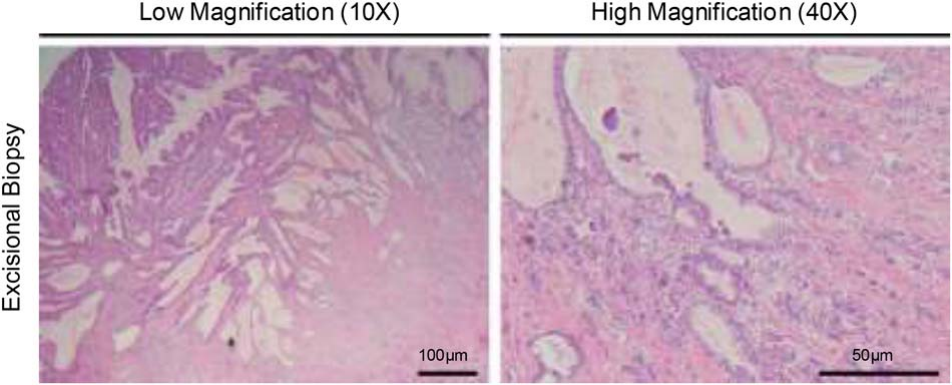
Hematoxylin and eosin staining of the resected specimen showing a moderately to poorly differentiated adenocarcinoma.

Given the patient’s anemia, melena and small bowel thickening, the patient was referred for upper and lower endoscopies, neither of which revealed the etiology of the patient’s symptoms. A pill endoscopy was also performed to evaluate the small intestine, though this was also unremarkable. The patient therefore underwent a double balloon enteroscopy, which was significant for a 3 cm small bowel mass located in the distal jejunum/proximal ileum (Fig. 2). The mass was biopsied and concerning for an adenocarcinoma. As a subsequent staging workup was negative for distant metastasis, the patient was taken for an exploratory laparotomy and small bowel resection. At the time of surgery, it was noted that the small bowel mass was located within an undiagnosed Meckel’s diverticulum (Fig. 3). The resected specimen was sent to pathology, which was significant for a moderately to poorly differentiated adenocarcinoma arising from a small bowel diverticulum, which was invading through the muscularis propria and into subserosal soft tissue with lymphovascular invasion (Fig. 4). The patient was therefore diagnosed with a Stage IIB (T4, N0) small bowel adenocarcinoma. After resection, the patient’s post-operative course was unremarkable, and he was discharged home on post-operative day 3. He was referred to medical oncology and is now receiving adjuvant capecitabine with oxaliplatin. As the patient’s resected specimen displayed negative margins, after completing the standard of care chemotherapy protocol, he will undergo active surveillance in accordance with the National Comprehensive Cancer Network guidelines.

## DISCUSSION

Malignant transformation of a Meckel’s diverticulum is extremely rare, affecting only 0.5–3.2% of symptomatic patients [8]. As the lining of the vitelline duct contains multipotent stem cells that would ordinarily give rise to any number of GI tissues [9], these cells can give rise to any number of cancer types once trapped in a Meckel’s diverticulum [5, 6]. Though carcinoid tumors are dominant, there are also reports of Meckel’s diverticulum-associated leiomyosarcomas, lymphomas, melanomas, papillary mucinous neoplasms and adenocarcinomas [5]. Meckel’s diverticulum-associated adenocarcinoma is believed to arise from this heterotopic tissue that includes pancreatic tissue, duodenal, jejunal, colonic and gastric mucosa [10]. Though this is generally well accepted, the underlying cause is poorly understood. Though some have suggested a potential role for *Helicobacter pylori*, this is unclear and warrants further study [5].

As Meckel’s diverticulum-associated adenocarcinoma can be so varied, pre-operative diagnosis remains a significant challenge. The presenting symptoms of malignancy related to a Meckel’s diverticulum are varied and often vague. Though there is no single pathognomonic sign, acute symptoms, including GI bleeding or perforation, or chronic symptoms such as anemia, obstruction or unintended weight loss can both be suggestive. However, at present, the majority of these tumors are diagnosed at an advanced stage, largely explaining their dismal prognosis. Additionally, there lacks consensus regarding the best treatment for adenocarcinomas related to a Meckel’s diverticulum. Treatment generally involves a diverticulectomy with primary small bowel anastomosis and appendectomy, though more extensive procedures may be indicated [5].

Hence, there is a clear and unmet need to better understand the warning signs of patients with Meckel’s diverticulum-related malignancies in order to facilitate early detection and improve clinical outcomes. Though cases such as ours serve as an important reminder of the potential of malignancy, however, at present it remains unclear whether a Meckel’s diverticulum should be removed prophylactically when found incidentally. This is fairly controversial. For example, a 1976 study argued that roughly 800 incidental diverticulectomies would be required to save a single life due to an associated malignancy. However, they also raised the important point of an approximate 8% rate of complication, and a mortality rate of 1.2%, suggesting that the risks outweigh the benefit for most patients [11]. As surgical techniques have advanced considerably in the decades since this work was published, this should be revisited. More recently, a group of investigators recommended a case-by-case approach for incidental diverticulectomy, taking into account select warning signs described above, as well as other symptoms favoring resection including a young age or the presence of other symptoms related to the Meckel’s diverticulum [12]. Hence, this should be taken into consideration when treating a patient with a known Meckel’s diverticulum, and the potential benefits and risks of operation discussed with the patient.

## CONFLICT OF INTEREST STATEMENT

The authors have no conflicts to disclose.

## FUNDING

DRP is supported by National Institutes of Health F30CA236031.
